# Changes in Televisit Modalities Due to the COVID Pandemic in Chile: A Comparison of Patient Satisfaction

**DOI:** 10.7759/cureus.55078

**Published:** 2024-02-27

**Authors:** Freddy Constanzo, Luis Benavides, Jorge Garcés, Rodrigo Villalobos, Mery Marrugo, Katia Kuzmanic, Ramón Caamaño, Lorena Peña, Cesar Silva, Cristobal Alvarado

**Affiliations:** 1 Adult Neurology, School of Medicine, Universidad Católica de la Santísima Concepción, Concepción, CHL; 2 Neurology, Hospital Las Higueras de Talcahuano, Talcahuano, CHL; 3 Telemedicine and Teleprocess, Hospital Las Higueras de Talcahuano, Talcahuano, CHL

**Keywords:** medical specialties, telemedicine, covid-19 pandemic, televisit, assisted televisit

## Abstract

Background: The coronavirus disease 2019 (COVID-19) pandemic has impacted healthcare guidelines and modalities of patient consultation worldwide. The frequent cycles of quarantine confinement in Chile have caused mobility restrictions for patients and physicians, forcing the Hospital Las Higueras de Talcahuano (HHT) to replace the assisted televisit modality with a more classic televisit program. Here we have described if this change in televisit modality and type of outpatient may have impacted patients’ satisfaction.

Methods: The patient’s perception of satisfaction was evaluated through self-administered survey questionnaires previously validated in Spanish. Cohorts were grouped according to the following two relational models: (i) assisted televisit, 503 neurology patients from 2018 to 2019, and (ii) televisit, 831 patients from different specialties treated during 2020. Perception of satisfaction was compared by gender, age, and type of televisit, and internal consistency (Cronbach alpha) and reliability (factorial analysis of principal components) were assessed. Finally, we compared the patient satisfaction of both modalities.

Results: Questionnaires showed excellent internal consistency; all items showed point biserial correlations greater than 0.30. Assisted televisit and televisit cohorts comprised 64.2% and 67.6% females, respectively, and patients under the age of 65 years were 62.2% and 75%, respectively. Assisted televisit patients showed very high 94.4% (n=475) and high 5.2% (n=26) satisfaction levels, while televisit patients showed very high 22.3% (n=185), high 63.9% (n=531), and moderate 13.1% (n=109) satisfaction levels; this difference was statistically significant at p<0.001.

Conclusion: Lower perception of satisfaction due to the change in televisit relational modality underscores the importance of primary care professionals who support the specialist in the assisted televisit model. However, the televisit modality showed high patient satisfaction and suggested that this modality can be a plausible alternative according to each location's reality. The results of this study indicate that both assisted televisit and televisit contribute to delivering an integrative solution that helps to alleviate the system's fragmentation.

## Introduction

Chile is located in the extreme south of the American continent, with a population of 19,678,363 inhabitants according to the latest census data [1]. As with any other country on the planet, the coronavirus disease 2019 (COVID-19) pandemic has impacted healthcare guidelines and modalities of patient consultation to face the challenges of different degrees of confinement during quarantine periods [2-4]. In 2020, the country ranked relatively high in worldwide COVID-19-related morbidity [5] and mortality [2]. During the COVID-19 pandemic, the Chilean healthcare system increased the number of intensive care unit (ICU) beds from 1,550 (April 14, 2020), with an occupancy rate of about 64%, to 4,484 (June 29, 2021) ICU beds with an occupancy rate of about 94% [6]. In addition, the massive admittance of patients with COVID-19 symptoms adversely impacted outpatient treatment, leading to a decrease in the number of in situ consultation hours for outpatients with non-COVID-19 diseases. Before the COVID-19 pandemic, the waiting list for the first specialty consultation on December 31, 2019, was 1,653,555 patients [7]. On March 31, 2021, the waiting list for the first specialty consultation was 1,932,422 patients, with an increase in the waiting list for the first consultation of 16% [8].

The Chilean healthcare system is organized in hospitals, tertiary facilities housing inpatient programs and specialist physician outpatient consultations, and primary or secondary care facilities, where general practitioners manage most non-specialty related outpatient consultations. A close interfacility relationship allows the timely transfer of a patient from the primary/secondary care facility to the hospital for either an outpatient consultation with a specialist physician or their admittance as an inpatient [7,8]. The multiple changes to quarantine confinement implemented throughout the COVID-19 pandemic have hindered patient and physician mobility and, thus, the flow of patients between the different levels of healthcare facilities. The Hospital Las Higueras de Talcahuano (HHT), a facility dependent on the Servicio de Salud de Talcahuano (SST), Ministry of Health, and located in the south of the country, is not strange to this reality. Among the several measures needed to overcome the limited bed availability and decreased time for outpatient consultation, televisit emerged as an opportunity to remotely treat outpatients, reducing the burden of the HHT personnel and the likelihood of COVID-19 contagion for the patients. Therefore, the HHT modified its outpatient programs to implement televisit for all health specialties.

Televisits can be implemented through several modalities, each with pros and cons regarding the relationship between the patient and the remote physician. In the HHT, the following two televisit models have been implemented, both of them coordinated by the Teleprocess Unit (TPU). First, a televisit modality, in which patients connect to the remote specialist on their own (usually from their homes); and second, an assisted televisit modality, in which patients connect, at the primary/secondary care facility or from their home, to the remote specialist physician, in the presence of a general practitioner acting as an in situ assistant to the specialist. The SST pioneered the latter model in Chile, with a teleneurology program implemented in March 2015, allowing adult neurological patients to be managed for their first consultation [8]. This assisted televisit program runs in a synchronous mode, with the outpatient consulting with a general practitioner in the primary/secondary care facility; the latter professional has been trained explicitly for this purpose and helps to connect the outpatient with a neurologist at the HHT via High Definition Television (HDTV) videoconference [7]. The HHT teleneurology program has successfully allowed the remote care of outpatients at local primary and secondary facilities. It has been shown to improve access to medical care by neurologist specialists by exhibiting a high level of user satisfaction while decreasing the waiting time for first consultation and providing timely follow-up appointments in different neurologic diseases [7-9].

Although the assisted televisit model has been successful in the HHT, this modality relies on patient mobility to secondary and tertiary healthcare facilities, which was hindered by frequent cycles of different degrees of quarantine confinement due to the COVID-19 pandemic. Therefore, the HHT was forced to move away from the assisted televisit modality in favor of a more classic televisit program to overcome both outpatient and physician quarantine-derived mobility restrictions [10,11]. In the latter modality, the medical specialist contacts patients directly from the HHT through an electronic device, such as a smartphone or computer (usually at home). The televisit modality has been previously shown to be successful in highly developed regions; however, this modality appears to be less accepted by communities in underdeveloped or developing regions [12-14]. Although most likely multifactorial, causes for community acceptance of the televisit or assisted televisit modalities have yet to be elucidated. The modality change in the HHT teleneurology program, from an assisted televisit consultation to a televisit one during the COVID-19 pandemic, provides an opportunity to evaluate potential differences in patient satisfaction between these two televisit modalities.

Therefore, we set out to investigate the patients' satisfaction by validating our previously published survey for the teleneurology program with outpatients from different medical specialties who were consulted through the televisit modality in 2020 [9]. Once the survey was validated, we compared the televisit patient satisfaction results with the assisted televisit modality implemented in 2019 [7]. The data described in this study was discussed in the context of factors that allowed the populations to prefer one modality in televisit [15].

This article, which compares both televisit modalities, was previously published as a preprint in The Lancet on August 12, 2021 [15].

## Materials and methods

Telemedicine care modalities

**Figure 1:**
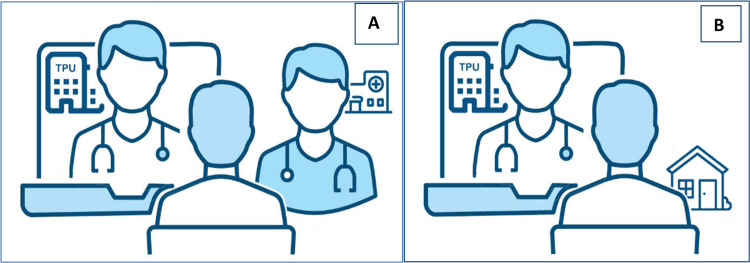
Type of attention: assisted televisit (A) and televisit (B). Representation of the assisted televisit (A) and televisit (B) used before and during the COVID-19 pandemic, provided by Teleprocess Unit (TPU). The image is original to the authors of this study.

This retrospective analytical observational cohort study compares the following two televisit relational models: (i) assisted televisit and (ii) televisit. Assisted televisit is a relational model already published by Constanzo et al. [7]. TPU at HHT connects the specialist doctor who is in the hospital with the patient and a general medical practitioner who is in the local primary/secondary health care service (Figure 1A). A general medical practitioner in the local primary care service accompanies the patient in this modality. The second model, televisit, was implemented in the HHT by the TPU due to the COVID-19 pandemic-derived confinement of patients in their homes. In this model, the specialist contacts the patient directly through electronic devices without the assistance of another health professional from the primary care service (Figure 1B).

Type of study

The sampling was performed using a proportion comparison formula for the retrospective analytical observational cohort study, which was calculated considering a risk (α) of 5%, a confidence level (1-α) of 95%, a statistical power (1-β) of 80%, a prevalence value of the assisted teleconsultation group of 74%, a prevalence value of the group with teleconsultation of 50%, an expected proportion of losses of 10%, finally being constituted by 70 individuals in each study group. All patients attended by assisted televisit and televisit modality were included in this study. Therefore, no sampling was required.

Patient cohorts

Patient cohorts were divided according to the following televisit models: (i) assisted televisit, corresponding to a cross-sectional study of 503 patients of the HHT Teleneurology program, treated by the assisted televisit modality during the years 2018-2019, and (ii) televisit, corresponding to a cross-sectional study of a cohort of 831 patients treated during 2020 in the HHT Telemedicine program (assisted by the TPU) from different medical specialties, such as bronchopulmonary, cardiosurgical, cardiology, endocrinology, gastroenterology, geriatric, hematology, internal medicine, physical medicine, nephrology, neurology, oncology, otorhinolaryngology, rheumatology, and others.

Inclusion criteria

Participants of the assisted televisit cohort had to meet the inclusion criteria described in the study by Constanzo et al. [7]. The inclusion criteria are as follows: (i) consent to remote care, including new appointments and controls; (ii) have an internet connection and at least a computer, a tablet, or a smartphone with a camera; (iii) present a pathology not requiring emergency care and are stable; (iv) be of legal age and mentally competent, according to the Chilean Law (Bill 28584, Article 28). The Scientific Ethics Committee of the Servicio de Salud Metropolitano Central, Ministry of Health, approved the research protocol. All participants signed a written informed consent.

Survey design assisted televisit and televisit

Two essentially identical surveys were employed to evaluate the patient’s perception of satisfaction. The assisted televisit survey estimated the patient perception of satisfaction using the questionnaire designed and constructed in Spanish, consisting of a total of 23 questions with closed responses on a five-point Likert scale (totally disagree, disagree, neither agree nor disagree, agree, and totally agree), which was previously validated by Constanzo et al. [7]. The survey questionnaire, with a maximum score of 115 points, was graded in terms of satisfaction as follows: very low (under or equal to 23 points), low (24-46 points), moderate (47-69 points), high (70-92 points), and very high (93-115 points). The televisit survey was identical to the previous one, except for excluding questions related to the general medical practitioner from the primary health center (questions 13, 17, 22, and 23), as this professional is absent from this model. Following this adaptation, the survey consisted of 19 questions with closed responses on a five-point Likert scale (totally disagree, disagree, neither agree nor disagree, agree, and totally agree). The survey questionnaire, with a maximum score of 95 points, was graded in terms of satisfaction as follows: very low (under or equal to 19 points), low (20-38 points), moderate (39-57 points), high (58-76 points), and very high (77-95 points). Both questionnaires were self-administered to safeguard the anonymity of the study participants (Table 1).

**Table 1:** Patient cohort description of assisted televisit and televisit cohort. Patient satisfaction was assessed for assisted televisit and televisit by the Teleneurology Unit of the Hospital Las Higueras de Talcahuano.

Telemedicine user satisfaction survey								
Age:				Gender:				
Mark with an “x” the preferred alternative								
N	Assisted televisit	Televisit	Strongly disagree		In disagreement	In disagreement	Agree	Totally agree
			1		2	3	4	5
1	I am satisfied with the medical care received during my telemedicine appointment.	I am satisfied with the medical care received during my telemedicine appointment.						
2	My family is satisfied with the medical care received during my telemedicine appointment.	My family is satisfied with the medical care received during my telemedicine appointment.						
3	Telemedicine helps me to be aware of my health status.	Telemedicine helps me to be aware of my health status.						
4	Telemedicine helps me to know how to improve my health status.	Telemedicine helps me to know how to improve my health status.						
5	Telemedicine allows me to better comply with the specialist doctor recommendations and indications.	Telemedicine allows me to better comply with the specialist doctor recommendations and indications.						
6	I was comfortable talking to the specialist doctor through a camera and a microphone.	I was comfortable talking to the specialist doctor through a camera and a microphone.						
7	Talking to my specialist doctor through a camera and a microphone, was as effective as doing it in person.	Talking to my specialist doctor through a camera and a microphone, was as effective as doing it in person.						
8	It was easy for me to explain my health condition to my specialist doctor during my telemedicine appointment.	It was easy for me to explain my health condition to my specialist doctor during my telemedicine appointment.						
9	My specialist doctor has identified my health problem during my telemedicine appointment.	My specialist doctor has identified my health problem during my telemedicine appointment.						
10	I have been informed of my right to privacy regarding my personal and medical information that was accessed during my telemedicine appointment.	I have been informed of my right to privacy regarding my personal and medical information that was accessed during my telemedicine appointment.						
11	I trust that my personal information and privacy will be protected after my telemedicine appointment.	I trust that my personal information and privacy will be protected after my telemedicine appointment.						
12	Image and sound qualities were adequate to talk to my specialist doctor.	Image and sound qualities were adequate to talk to my specialist doctor.						
13	The general doctor who accompanied me in person helped me during my telemedicine appointment.							
14	My telemedicine appointment was helpful.	My telemedicine appointment was helpful.						
15	The time I have to wait for scheduling the appointment with a specialist is short if it is a telemedicine appointment.	The time I have to wait for scheduling the appointment with a specialist is short if it is a telemedicine appointment.						
16	I would rather schedule a telemedicine appointment since it is easier to go to the local family clinic than to the hospital.	I would rather schedule a telemedicine appointment since it is easier to go to the local family clinic than to the hospital.						
17	I would rather schedule a telemedicine appointment since it is cheaper to go to the local family clinic than to the hospital.							
18	For my future medical care, I would rather schedule telemedicine appointments.	For my future medical care, I would rather schedule telemedicine appointments.						
19	My specialist doctor was able to answer my questions during my telemedicine appointment.	My specialist doctor was able to answer my questions during my telemedicine appointment.						
20	My specialist doctor was engaged in solving my health problem during my telemedicine appointment.	My specialist doctor was engaged in solving my health problem during my telemedicine appointment.						
21	I trust the instructions of my specialist doctor during my telemedicine appointment.	I trust the instructions of my specialist doctor during my telemedicine appointment.						
22	The general practitioner who accompanied me in person during the telemedicine appointment was able to answer my questions.							
23	The general practitioner who accompanied me in person during the telemedicine appointment could answer the questions made by my specialist doctor							

Survey evaluation and statistical analysis

A descriptive analysis of the normality of the sample (Kolmogorov-Smirnov) was conducted. The internal consistency was evaluated by Cronbach's alpha test, which suggests the following scale for alpha coefficients: excellent (>0.9), good (>0.8), acceptable (>0.7), questionable (>0.6), poor (>0.5), and unacceptable (<0.5) [7]. Difficulty and discrimination of the instrument were evaluated by the index of difficulty and specific biserial correlation, respectively. To compare the means of both surveys with a non-normal distribution, we used the Mann-Whitley U test for independent samples. All analyses were carried out using SPSS version 25.0 (Armonk, NY: IBM Corp.). Statistical significance was established at p<0.05.

## Results

Patient cohort description

The assisted televisit cohort (N=503) consisted of 64.2% (N=323) females; 62.2% (N=313) of patients were under 65 years of age. On the other hand, the televisit cohort (N=831) consisted of 67.6% (N=562) females; 75% (N=623) of patients were under 65 years of age (Table 2).

**Table 2:** Specialties that participate in the televisit modality. Patients of assisted televisit (N=503) and televisit (N=803) modalities were categorized by gender and age.

Variables	Assisted televisit		Televisit	
	N	%	N	%
Gender				
Male	180	35.8	269	32.4
Female	323	64.2	562	67.6
Total	503	100.0	831	100.0
Age				
Under 65 years	313	62.2	623	75.0
Over 65 years	190	37.8	208	25.0
Total	503	100.0	831	100.0

All participants of the assisted televisit cohort were neurological patients. Those of the televisit cohort consulted different medical specialties as follows: endocrinology 20% (N=165), otorhinolaryngology 15% (N=126), bronchopulmonary 15% (N=122), internal medicine 11% (N=91), neurology 8% (N=68), gastroenterology 7% (N=56), cardiology 6% (N=46), hematology 5% (N=39), cardiosurgery 3% (N=26), others 3% (N=23), rheumatology 3% (N=21), physical medicine and rehabilitation 2% (N=19), geriatrics 1% (N=11), oncology 1% (N=10), and nephrology 1% (N=8) (Table 3).

**Table 3:** Televisit outpatients medical specialty. Televisit patients were categorized by medical specialty (N=831). All patients of assisted televisit were neurology patients.

Medical specialty	Frequency	Percentage
Endocrinology	165	20%
Otorhinolaryngology	126	15%
Bronchopulmonary	122	15%
Internal medicine	91	11%
Neurology	68	8%
Gastroenterology	56	7%
Cardiology	46	6%
Hematology	39	5%
Cardiosurgery	26	3%
Others	23	3%
Rheumatology	21	3%
Physical medicine and rehabilitation	19	2%
Geriatrics	11	1%
Oncology	10	1%
Nephrology	8	1%
Total	831	100%

**Figure 2:**
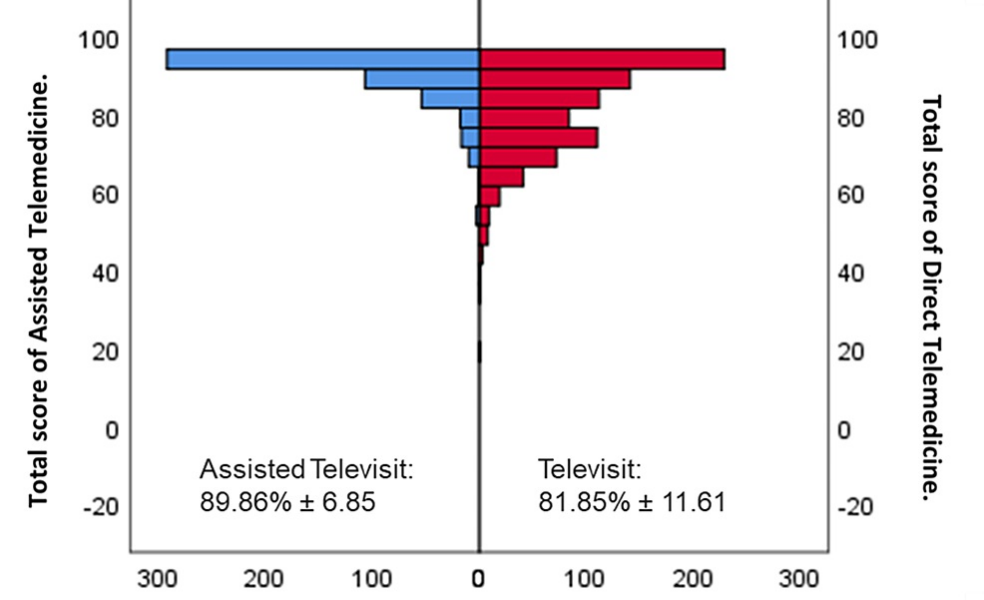
Type of attention comparison. We compare the user satisfaction between assisted televisit (left-blue, n=503) and televisit (right-red, n=831). The Mann-Whitley U test for independent samples showed significantly higher user satisfaction in the assisted televisit attention (p=0.000).

In terms of the perception of satisfaction with each model, different patterns of satisfaction arose. Assisted televisit patients showed a very high level of satisfaction at 94.4% (N=475), which increased by 5.2% (N=26) (Table 4). In comparison, televisit patients showed very high satisfaction at 22.3% (N=185), high satisfaction at 63.9% (N=531), and moderate satisfaction at 13.1% (N=109) (Table 5). Comparison of participant satisfaction in both cohorts by gender and age did not result in significant differences at p<0.05 (Kolmogorov Smirnov's p<0.05; data not shown). To properly compare the satisfaction perception of the two cohorts, we did not consider questions 13, 17, 22, and 23 from the assisted televisit questionnaire. Assisted televisit showed significantly higher patient satisfaction (89.86%±6.85) than televisit cohorts (81.85%±11.61) (p<0.001 Mann-Whitley U test) (Figure 2).

**Table 4:** Patient satisfaction of all outpatients attended by assisted televisit (N=503).

Variables	Total	
	N	%
User satisfaction		
Very low satisfaction (≤23 points on satisfaction scale)	0	0%
Low satisfaction (24-46 points on satisfaction scale)	0	0%
Moderate satisfaction (47-69 points on satisfaction scale)	2	0,4%
High satisfaction (70-92 points on satisfaction scale)	26	5,2%
Very high satisfaction (93-115 points on satisfaction scale)	475	94.4%
Total	503	100%

**Table 5:** Patient satisfaction of all outpatients attended by televisit (N=831).

Variables	Total	
	N	%
User satisfaction		
Very low satisfaction (≤19 points on satisfaction scale)	1	0.1%
Low satisfaction (20-38 points on satisfaction scale)	5	0.6%
Moderate satisfaction (39-57 points on satisfaction scale)	109	13.1%
High satisfaction (58-76 points on satisfaction scale)	531	63.9%
Very high satisfaction (77-95 points on satisfaction scale)	185	22.3%
Total	831	100%

Survey evaluation

The reliability analysis of the questionnaire showed that internal consistency was high, with a Cronbach's alpha of 0.90 and 0.94 for assisted televisit and televisit, respectively (Table 6). Every item showed Cronbach's alpha values around 0.9. Means of patient satisfaction scores were 104.4±0.12 points and 77.55±0.21 for assisted televisit and televisit, respectively. The elimination of any of the questionnaire items led to maintaining its reliability as very high (Cronbach's alpha>0.9), regardless of the identity of the removed item. The difficulty of the instrument's questions was assessed using the difficulty index. None of the items showed a difficulty index lower than 0.10, confirming high discrimination indexes (Table 6).

**Table 6:** Survey validation for televisit patients. Descriptive data of the total population by mean per question, difficulty, mean when the question is deleted, reliability analysis (a-Cronbach), and biserial correlation of the patient satisfaction survey, used in assisted televisit (N=503) and televisit (N=831).

N	Variables	Assisted televisit (N=503)					Televisit (N=831)				
		Mean per question	Difficulty	Biserial Correlation	Mean when the question is deleted	α when the question is deleted	Mean per question	Difficulty	Biserial Correlation	Mean when the question is deleted	α when the question is deleted
						0.9					0.94
1	I am satisfied with the care received in Telemedicine.	4.83	0.97	0.63	104.22	0.9	4.47	0.89	0.7	77.39	0.93
2	My family is satisfied with the care received in Telemedicine.	4.44	0.89	0.4	104.62	0.9	4.28	0.86	0.66	77.57	0.93
3	Telemedicine helps me to know my state of health.	4.75	0.95	0.54	104.3	0.9	4.16	0.83	0.71	77.7	0.93
4	Telemedicine helps me know how to improve my health status.	4.71	0.94	0.55	104.34	0.9	4.18	0.84	0.69	77.67	0.93
5	Telemedicine allows me to better follow the recommendations and indications of my specialist doctor.	4.79	0.96	0.58	104.27	0.9	4.28	0.86	0.7	77.57	0.93
6	I felt comfortable talking to my specialist doctor through a camera and a microphone.	4.71	0.94	0.54	104.34	0.9	4.49	0.9	0.68	77.36	0.93
7	Talking to my specialist doctor. through a camera and a microphone was as effective as in person.	4.58	0.92	0.69	104.48	0.89	3.89	0.78	0.74	77.97	0.93
8	During my Telemedicine care it was easy for me to explain my health problem to my specialist doctor.	4.67	0.93	0.61	104.38	0.9	4.4	0.88	0.71	77.45	0.93
9	My specialist doctor has identified my health problem through Telemedicine.	4.7	0.94	0.53	104.36	0.9	4.22	0.84	0.67	77.64	0.93
10	I have been informed of my right to privacy of my personal and medical information included in Telemedicine.	4.68	0.94	0.22	104.37	0.91	4.05	0.81	0.38	77.8	0.94
11	I trust that my personal information and privacy will be protected after my attention by Telemedicine.	4.81	0.96	0.54	104.24	0.9	4.39	0.88	0.62	77.47	0.93
12	The quality of the image and sound were adequate to talk to my specialist doctor.	4.76	0.95	0.36	104.3	0.9	4.5	0.9	0.58	77.35	0.93
13	The general doctor who accompanied me in person helped me during my Telemedicine consultation.	4.88	0.98	0.5	104.17	0.9	-	-	-	-	-
14	My attention by Telemedicine was helpful to me.	4.84	0.97	0.74	104.21	0.89	4.45	0.89	0.82	77.41	0.93
15	The time with a specialist is faster by Telemedicine.	4.74	0.95	0.49	104.31	0.9	4.21	0.84	0.54	77.65	0.94
16	I prefer Telemedicine because it is easier to go to the doctor's office than to go to the hospital.	4.71	0.94	0.61	104.34	0.9	4.25	0.85	0.57	77.6	0.93
17	I prefer Telemedicine because it is cheaper to go to the office than to go to the hospital.	4.56	0.91	0.49	104.5	0.9	-	-	-	-	-
18	For my future controls I will prefer to continue using Telemedicine.	4.56	0.91	0.49	104.5	0.9	18	For my future controls, I will prefer to continue using Telemedicine.	4.56	0.91	0.49
19	My specialist doctor was able to answer my questions through Telemedicine	4.87	0.97	0.63	104.19	0.9	19	My specialist doctor was able to answer my questions through Telemedicine.	4.87	0.97	0.63
20	My specialist doctor showed concern in solving my health problem during Telemedicine care.	4.84	0.97	0.52	104.21	0.9	20	My specialist doctor showed concern in solving my health problem during Telemedicine care.	4.84	0.97	0.52
21	I trust the instructions of my specialist doctor during my Telemedicine care.	4.87	0.97	0.66	104.18	0.9	21	I trust the instructions of my specialist doctor during my Telemedicine care.	4.87	0.97	0.66
22	The general practitioner who accompanied me in person during the Telemedicine service was able to answer my questions.	4.86	0.97	0.55	104.19	0.9	22	The general practitioner who accompanied me in person during the Telemedicine service was able to answer my questions.	4.86	0.97	0.55
23	The general practitioner who accompanied me in person during the Telemedicine care could answer the questions of my specialist doctor.	4.89	0.98	0.54	104.16	0.9	23	The general practitioner who accompanied me in person during the Telemedicine care could answer the questions of my specialist doctor.	4.89	0.98	0.54

To properly compare the satisfaction perception of the two cohorts, we did not consider questions 13, 17, 22, and 23 from the Assisted televisit questionnaire. Assisted Televisit and Televisit cohorts showed mean satisfaction of 89.86%±6.85 and 81.85%±11.61, respectively (p<0.001, Kolmogorov Smirnov's p<0.05).

Survey evaluation

The reliability analysis of the questionnaire showed that internal consistency was high, with a Cronbach's alpha of 0.90 and 0.94 for assisted televisit and televisit, respectively (Table 6). Every item showed Cronbach's alpha values around 0.9. Means of patient satisfaction scores were 104.4±0.12 points and 77.55±0.21 for assisted televisit and televisit, respectively. The elimination of any of the questionnaire items led to maintaining its reliability as very high (Cronbach's alpha >0.9), regardless of the identity of the removed item. The difficulty of the instrument's questions was assessed using the difficulty index. None of the items showed a difficulty index lower than 0.10, confirming high discrimination indexes (Table 6).

## Discussion

Televisit arises as an attractive tool to address some of the deficits in timely access to healthcare for patients from different settings [16]. Telemedicine can provide health care to new and controlled patients from a vast geographical area, contributing to the continuity of care [17,18] and the operational principle of system sufficiency and minimum social security benefits [19]. In Chile, the HHT has pioneered the implementation of a teleneurology program since 2015, with the ambitious goal of increasing access to adult patients through remote neurological consultation [7]. Since its inception, the TPU at HHT has employed the assisted televisit model [8,9]. However, the COVID-19 pandemic forced the switch to a televisit model in the Neurology Unit, and the further widening to other medical specialties, which did not previously have a telemedicine model. In fact, after the model switch, the TPU started with three specialties (cardiology, heart surgery, and neurology) and currently handles 15 different medical specialties. Moreover, endocrinology, otorhinolaryngology, and bronchopulmonary seamlessly adopted the televisit model, which now covers 50% of their patient population. Remarkably, due to its simplicity and low human resources and materials cost, it is currently the televisit model used in the HHT for all medical specialties.

In the present study, we compared the patient’s perception of satisfaction between assisted televisit and televisit models for the first time in Chile. Statistical analyses of data from this study showed the designed questionnaires' high reliability and internal consistency. In addition to the previously reported data, biserial correlation analysis showed that all selected questionnaire items were suitable for surveys used in both patient cohorts [7]. In agreement with our previous findings, the present study indicates that neither telemedicine intervention hinders the development of the link between patient and their specialists [7]. In addition, there are no significant differences in patients’ satisfaction by gender or age. Telemedicine might be helpful to the population, regardless of age, as long as they are digitally able.

Our analysis showed that assisted televisit displayed significantly higher satisfaction than the televisit model. This underlines the importance of the primary care professionals to support the specialist during the televisit to achieve greater user satisfaction in the public health system. This makes the assisted televisit model at the HHT one of many high standards of care delivered daily [16,20]. Although lower than the assisted televisit model, televisit still displayed high patient satisfaction. Thus, it can be considered for exceptional situations, such as following patients: (i) located in remote areas with little or no access to primary or secondary health services or inaccessible for home visits, (ii) in disaster areas, (iii) with severe mobility limitation, (iv) in overloaded health centers, or (v) isolated/quarantined patients, as is the case in the COVID-19 pandemic.

This study is based on data from participants grouped in cohorts by convenience sampling. Participants from each cohort were recruited at different times - the assisted televisit during 2015-2019 and the televisit cohort during 2020. Since the latter cohort had an additional stressor, i.e., a quarantine confinement during a very publicized pandemic, lower satisfaction scores may be explained, at least in part, because of a higher psychological stress on patients rather than the actual televisit perception [21]. Conversely, the assisted televisit cohort of patients belonged to only one specialty (adult neurology) with over five years of experience with telemedicine programs, while the televisit cohort belonged to 15 different specialties that did not have prior experience in this field. Therefore, the difference in professional team experience with the televisit paradigm may have impacted the perception of satisfaction by their patients. Nevertheless, evidence suggests that patients' perception of satisfaction with televisit programs varies according to their needs [17]. The consensus among specialists [17] is that there are following three pillars required to achieve high levels of user satisfaction with a televisit program: (i) good connectivity to achieve a good audio and video connection, (ii) readily availability of electronic medical records, and (iii) of a patient-centered care system [18]. The present study underscores how relevant the third pillar is for the perception of satisfaction of the patient. The population served through the assisted televisit model has greater user satisfaction than a more classical one. We propose that such greater satisfaction is likely due to a patient’s better understanding of and access to all the indications the specialist physician gives through reinforcement and management by the general practitioner who accompanies the patient in situ during the televisit [8]. This modality allows for several advantages for the patient, including (i) the patient is better appraised of their diagnosis and treatment by two physicians instead of one, reinforcing the clinical messages [7,8]; (ii) in most cases, there is an already ongoing rapport between the patient and the general practitioner, which in turn, facilitates and strengthens one of the patients with the specialist [7,8]; and (iii) since the general practitioner works in a healthcare institution that covers the geographical area where the patient lives in, this professional can better perceive the patient’s livelihood reality, facilitating the specialist-patient dialogue [7,8]. Furthermore, the assisted televisit model generates a space for continuous collaboration between tertiary and primary healthcare, highlighting the importance of teamwork that contributes to overcoming the fragmentation evidenced in the Chilean health system and other latitudes through an integrative solution that reinforces a patient-centered praxis instead of an institutional-centered one [20].

The HHT TPU has pioneered the assisted televisit modality in the region, and the hurdles imposed by the COVID-19 pandemic have allowed for the widening of the specialty spectrum of telemedicine programs. In addition, there are still several landmarks to be accomplished in this area of healthcare - (i) to further widen the spectrum of specialties covered by the TPU, (ii) to educate the health community and patients in remote care, and (iii) to create a new digital hospital with new facilities. Overall, the high satisfaction scores in the present study indicate that patients in both the public and private healthcare systems would benefit by implementing telemedicine programs, particularly under the assisted model.

## Conclusions

We point out that we should consider a few relevant limitations in this study the survey and size bias.* *However, we conclude that the lower perception of patient satisfaction due to the change in televisit relational modality underscores the importance of primary care professionals who support the specialist in the assisted televisit model. However, the televisit modality showed high patient satisfaction and suggested that this modality can be a plausible alternative according to each location's reality. The results of this study indicate that both assisted televisit and televisit contribute to delivering an integrative solution that helps to alleviate the system's fragmentation.
